# A screening method for cervical myelopathy using machine learning to analyze a drawing behavior

**DOI:** 10.1038/s41598-023-37253-3

**Published:** 2023-06-20

**Authors:** Eriku Yamada, Koji Fujita, Takuro Watanabe, Takafumi Koyama, Takuya Ibara, Akiko Yamamoto, Kazuya Tsukamoto, Hidetoshi Kaburagi, Akimoto Nimura, Toshitaka Yoshii, Yuta Sugiura, Atsushi Okawa

**Affiliations:** 1 Department of Orthopedic and Spinal Surgery, Graduate School of Medical and Dental Sciences, Tokyo Medical and Dental University (TMDU), 1-5-45, Yushima, Bunkyo-ku, Tokyo, 113-8519 Japan; 2 Department of Functional Joint Anatomy, Graduate School of Medical and Dental Sciences, Tokyo Medical and Dental University (TMDU), 1-5-45, Yushima, Bunkyo-ku, Tokyo, 113-8519 Japan; 3 School of Science for Open and Environmental Systems, Graduate School of Science and Technology, Keio University, 3-14-1 Hiyoshi, Kohoku-ku, Yokohama-shi, Kanagawa, 223-8522 Japan

**Keywords:** Diseases, Health care, Medical research, Neurology, Signs and symptoms

## Abstract

Early detection of cervical myelopathy (CM) is important for a favorable outcome, as its prognosis is poor when left untreated. We developed a screening method for CM using machine learning-based analysis of the drawing behavior of 38 patients with CM and 66 healthy volunteers. Using a stylus pen, the participants traced three different shapes displayed on a tablet device. During the tasks, writing behaviors, such as the coordinates, velocity, and pressure of the stylus tip, along with the drawing time, were recorded. From these data, features related to the drawing pressure, and time to trace each shape and combination of shapes were used as training data for the support vector machine, a machine learning algorithm. To evaluate the accuracy, a receiver operating characteristic curve was generated, and the area under the curve (AUC) was calculated. Models with triangular waveforms tended to be the most accurate. The best triangular wave model identified patients with and without CM with 76% sensitivity and 76% specificity, yielding an AUC of 0.80. Our model was able to classify CM with high accuracy and could be applied to the development of disease screening systems useful outside the hospital setting.

## Introduction

Cervical myelopathy (CM) is characterized by stenosis of the cervical spine and may result in gait disturbance and hand clumsiness^1,2^. Patients with CM are often unaware of their condition due to the slow progression of symptoms. Therefore, by the time symptoms manifest and patients seek treatment, CM often progresses to a severe stage^3,4^. Patients with advanced CM often report an unstable gait^5,6^ and are prone to falls, which can lead to further spinal cord injuries or spine fractures^7^. Though the symptoms of CM can improve with early treatment, those reported in the advanced stages may be irreversible, likely resulting in poor treatment outcomes^8^. Thus, early detection and treatment of CM is desirable.

CM symptoms often begin with the upper extremities^9^, such as clumsiness of the hands, known as “myelopathic hand”^10^. The myelopathic hand is characterized by a loss of manual dexterity, which may lead to the patient struggling to write or button. Early detection of CM requires a physical examination by an experienced doctor or the use of objective evaluation methods, such as the 10-s grip and release test, and finger escape sign^10^. However, there are few established objective evaluation parameters for CM symptoms involving activities of daily living, such as difficulty writing or buttoning.

Recently, we focused on the writing behavior of patients with various diseases that cause writing disorders and developed a novel screening tool using machine learning that can identify carpal tunnel syndrome^11^. This method allowed for the screening of carpal tunnel syndrome by analyzing the changes in drawing pressure and trajectory accuracy resulting from sensory disturbances and atrophy of the thenar muscle. In the present study, we applied the previous method to writing disorders related to CM and developed a classification method for CM. Our method is intended to be used to develop a basic tool, comprised of a commercially available tablet and stylus pen, for CM screening outside the hospital setting. To the best of our knowledge, there have been no previous reports of tools using artificial intelligence to screen for CM by objectively analyzing writing movements. In this study, we hypothesized that the quantitative assessment of writing impairment using machine learning could be applied to CM detection. Therefore, this study aimed to examine whether the method we developed is a useful screening tool for CM.

## Results

Participant demographic characteristics are summarized in Table 1. No significant differences were noted in age or sex between the non-CM and CM groups. The severity of the patients’ CM is shown in Table 2; a relatively even distribution of patients with mild to severe disease was observed in this study.

**Table 1:** Participant demographic characteristics in the CM and non-CM groups.

	Non-CM group	CM group	p-value
Number of participants, N	66	38	
Age in years, median (IQR)	69 (59–73)	66 (57–76)	0.71
Sex (female), N (%)	38 (58)	15 (39)	0.07

CM, cervical myelopathy; IQR, interquartile range.

**Table 2:** Number of patients with cervical myelopathy demonstrating varying degrees of severity according to the JOA score.

Severity	JOA score	N
Mild	> 13	9
Moderate	9–13	18
Severe	< 9	11
Total		38

JOA, Japanese Orthopedic Association.

### Pressure analysis of the stylus tip and drawing time

Table 3 shows the results of the comparison of the three features in terms of average drawing pressure, smoothness of the pressure change, and drawing time. In all three shapes, the average value of drawing pressure was significantly lower in the CM group than in the non-CM group. The smoothness of the change in writing pressure for spirals was significantly lower in the CM group than in the non-CM group, while, for square and triangular waves there was no significant difference in that between the two groups. Regarding drawing time, there was no significant difference between the two groups for all shapes.

**Table 3:** Comparison of the three features between the CM and non-CM groups.

	Spiral		
	Non-CM group	CM group	p-value
DT (IQR) (seconds)	9.5 (8.0–11.7)	9.8 (8.6–11.2)	0.67
AP (IQR)	1.8 (1.3–2.3)	1.2 (0.8–1.7)	0.00077
SP (IQR)	− 102.8 (− 117.2, − 57.5)	− 116.3 (− 128.8, − 80.0)	0.01

	Square wave		
	Non-CM group	CM group	p-value
DT (IQR) (seconds)	5.6 (4.3–7.7)	5.8 (5.1–8.3)	0.28
AP (IQR)	1.5 (1.2–2.1)	1.0 (0.7–1.6)	0.00095
SP (IQR)	− 56.9 (− 70, − 41.4)	− 61 (− 80.5, − 45.3)	0.31

	Triangular wave		
	Non-CM group	CM group	p-value
DT (IQR) (seconds)	5.0 (3.8–7.0)	5.4 (4.4–7.5)	0.35
AP (IQR)	1.5 (1.2–2.1)	1.1 (0.7–1.6)	0.002
SP (IQR)	− 53.2 (− 67.0, − 34.7)	− 61.4 (− 74.2, − 43.1)	0.05

IQR, interquartile range; DT, drawing time; AP, average pressure; SP, SPARC (spectral arc length).

### CM classification using a support vector machine

**Figure 1:**
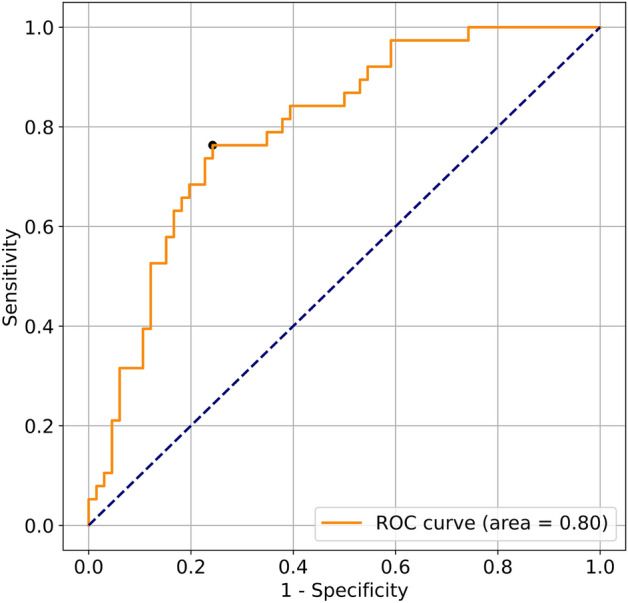
The receiver operating characteristic (ROC) curve for the triangular wave model. When the threshold is set to a point near the upper left of the graph, the sensitivity and specificity were 76% and 76%, respectively. The area under the curve was 0.80.

The results of the support vector machine (SVM) classification of each of the three shapes and their combinations are shown in Table 4. Of all models, the triangular wave model had the highest area und the curve (AUC, 0.80). For models that combined multiple shapes, those that included triangular waves had high accuracy. The receiver operating characteristic (ROC) curve of the best triangular wave model is shown in Fig. 1. When the threshold was set closest to the upper-left point on the ROC curve, the sensitivity and specificity were 76% and 76%, respectively.

**Table 4:** Area under curve of the support vector machine (SVM) models.

Shape	Sensitivity	Specificity	AUC
Spiral	0.66	0.52	0.65
Square	0.63	0.68	0.61
Triangular	0.76	0.76	0.80
Spiral + Square	0.71	0.61	0.68
Square + Triangular	0.68	0.73	0.75
Triangular + Spiral	0.74	0.80	0.78
All	0.63	0.89	0.77

AUC, area under the curve.

## Discussion

In this study, we developed a novel classification method for CM, focused on drawing behavior, using a commercially available tablet device and stylus pen. We recorded the participants’ drawing time and drawing pressure while they traced spiral, square, and triangular waves on a tablet. Using these data and a machine learning algorithm, we demonstrated high classification accuracy with 76% sensitivity, 76% specificity, and an AUC of 0.80. In previous reports, the 10-s grip and release test showed a sensitivity of 61% to 61.7% and an AUC of 0.74^12–14^, the finger escape sign showed a sensitivity of 48–55%^12,13^, and the deep tendon reflex change showed a sensitivity of 15–56%^15,16^. Our method had a higher sensitivity than that of conventional physical tests, indicating its usefulness as a screening tool.

There have been many reports on handwriting in neurological diseases^17–19^. Previous studies have examined the writing pressure and kinematic features in the spiral drawings of patients with Parkinson’s disease using diagnostic methods, which are also used to assess the severity of motor disorders in patients with other neurodegenerative diseases^20–23^. Combined machine learning-based diagnostic tools for Parkinson's disease have also been developed and have demonstrated high accuracy^24,25^. Furthermore, a highly accurate classifier has been reported for Alzheimer's disease, focusing on features related to the speed of handwritten signatures^26^. There have also been several reports of methods to detect dysgraphia in children^27,28^. A tablet-based diagnostic tool was developed to predict dysgraphia by focusing on the static, kinematic, pressure, and tilt features of writing behavior^29^. However, to the best of our knowledge, no studies have reported on methods that use machine learning to diagnose writing disorders in CM. This study is the first report of a simple screening method for CM based on a drawing task and using machine learning.

In CM, intrinsic hand muscle functions are decreased^30^ and there is a distinct spasticity of the hands^31^, which may lead to clumsiness. Thus, it is clinically plausible that the average drawing pressures will be lower overall. In terms of the drawing pressure smoothness indicated by spectral arc length (SPARC), the value for spiral waves was significantly lower in the CM group than in the non-CM group, indicating less smoothness, which is also a reasonable finding. On the other hand, both groups had greater values for square and triangular waves than for spirals, meaning that the writing pressures were smoother for these shapes. However, the SPARC values in the square and triangular waves were not significantly different between groups. The reason for this could be that these two figures are composed of several short lines, with short pauses in the middle, making it more difficult to distinguish smoothness compared to the spiral, which is written continuously over a longer period of time. Meanwhile, no significant difference was observed in the drawing time for all shapes. The participants were asked to write at their own preferred speed, with no instructions as to how fast they should write. Therefore, regardless of the group, some wrote quickly, while others wrote carefully and slowly. Different results might be observed if they were instructed to write as quickly as possible, and future studies are needed to verify this. For the SVM classification model, high accuracies were observed in the models with triangular waves. This may be because in a triangular wave, the tip of the stylus needs to be turned back in the opposite direction, which is difficult for patients with CM who have poorer hand control. In addition, previous reports investigating writing angles found that right-handers have biomechanical properties among the hand muscles that favor right ascending lines and hinder right descending lines^32,33^. A study also reported differences in speed and line length accuracy when older people drew right ascending and right descending lines^34^. In this study, all participants were right-handed, and the rightward triangular waves, which included both right ascending and right descending lines, could have made the difference more pronounced in patients with CM, possibly increasing the accuracy of distinguishing CM from non-CM.

The method presented in this report requires no special equipment other than a commercially available tablet device and stylus pen. It can be implemented not only in hospital settings but also in an out-of-hospital setting, such as at home. Although this was a cross-sectional study of pre-diagnosed CM patients and further prospective study is needed, this method may lead to techniques for the early detection of CM, prompting patients to visit spine specialists to confirm the diagnosis and receive early treatment. There have been several approaches for the early detection of CM outside the hospital setting. For example, web-based symptom checkers are widely used as diagnostic tools for CM. However, these tools are limited in their ability to accurately diagnose mild symptoms, and further optimization is needed^35^. A previous study reported a system to diagnose CM, using a non-contact sensor device and artificial intelligence to analyze hand grip and release, which may lead to early detection outside hospital settings^36^. Our study is novel in that we objectively analyzed writing behavior, a common activity of daily living. While it is currently necessary to use specific shapes, in the future, this method could be applied using unspecified shapes, such as when writing one’s name, and could lead to the development of a method that allows for unconscious screening in daily life.

This study has some limitations. First, we did not analyze other diseases that affect writing movements, such as carpal tunnel syndrome, cubital tunnel syndrome, or Parkinson's disease. We have already developed a classification method for carpal tunnel syndrome and plan to classify multiple diseases in our future work. Second, the participants in the CM group were only pre-operative patients, causing concern that there would be a higher proportion of severe cases. However, the fact that cases graded mild and moderate using the Japanese Orthopedic Association (JOA) scoring system were included suggests that this method could still be adapted for use as a screening tool. Third, we did not limit the level of spinal cord compression and or perform level-by-level comparative verification. The main purpose of this method was screening, and the correlation between the level of stenosis and the degree of impairment of writing movements needs to be studied separately. Fourth, this method does not have sufficient sensitivity to be used as a screening tool as of now. Although higher than conventional physical examination, higher sensitivity and accuracy are needed for use outside the hospital and in everyday life. However, the strength of this model is that it can be updated sequentially by adding cases, and we aim to bring the model closer to implementation as its accuracy improves in the future. For example, the addition of features related to sensation and strength has the potential to increase the model accuracy; these will be added to the analysis as the number of samples increases in the future.

In conclusion, we developed a novel classification method that provides the basis for a CM screening system using a machine learning algorithm-based analysis of drawing behavior. By integrating features related to drawing behavior, we obtained a model with high classification accuracy. Using only a commercially available tablet device and stylus pen, this method could be used for the development of disease screening systems, for in- and out-of-hospital settings, that would facilitate the early detection and treatment of CM.

## Methods

### Participants

In the present study, we enrolled 38 patients with CM (CM group) and 66 healthy volunteers (non-CM group) between September 2020 and September 2022. The CM group included preoperative patients with CM scheduled for cervical spine surgery at the Tokyo Medical and Dental University Hospital. CM was diagnosed by experienced spine surgeons based on physical and neurological examination and findings of cervical spine stenosis on magnetic resonance imaging. Patients were assessed for severity according to the JOA score for CM, which has a maximum score of 17, with lower scores indicating more severe disease. Based on past literature, the severity was divided into three grades: mild (JOA score higher than 13), moderate (9–13), and severe (lower than 9)^37^. The control group included patients who underwent total hip arthroplasty at the same hospital. The healthy volunteers had no symptoms indicating CM, such as clumsiness or numbness of the hands, or any previous history of a disease that may cause difficulty in using the hands. Furthermore, cervical spine X-rays, which are routinely used to evaluate the general alignment of the spine, were used to help rule out cervical disease by confirming the absence of findings such as degeneration or ossification of ligaments^36^. In both groups, patients were excluded from the analysis if they had a history of peripheral neuropathy, diabetes mellitus, Parkinson's disease, hydrocephalus, stroke, trauma to the upper extremities, or surgery of the cervical spine and upper extremities. These exclusion criteria were confirmed by several methods: an interview, a thorough electronic medical record check of the patient's history, and a physical examination by an experienced doctor. We also excluded individuals who could not perform the tasks described below due to poor eyesight.

This study was approved by the Medical Research Ethics Committee of Tokyo Medical and Dental University (#M2019-047) and performed in compliance with the Declaration of Helsinki. All patients provided informed consent prior to study participation, as approved by the ethical committee of the institution.

### Apparatus and writing task

**Figure 2:**
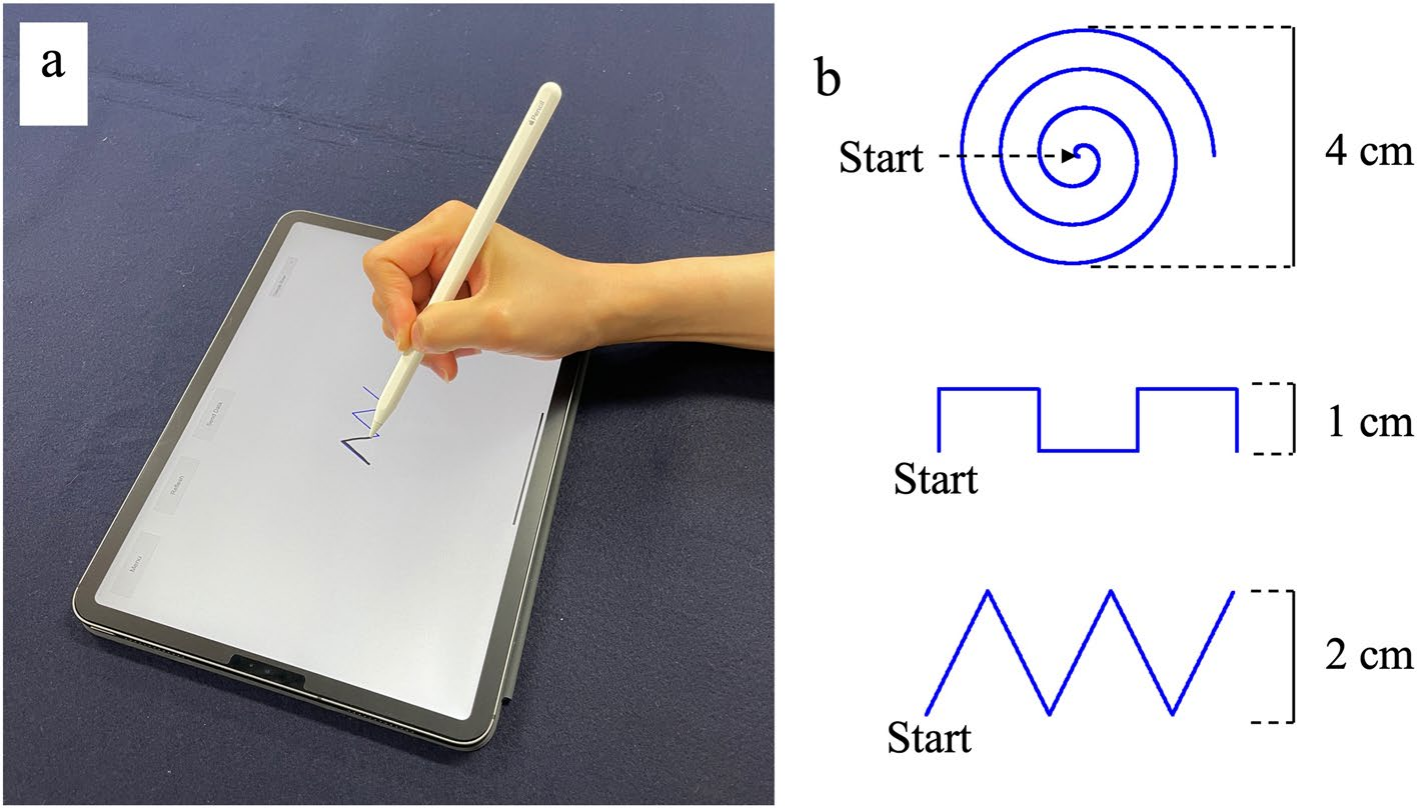
Measurement system and sample shapes. (**a**) Participants wrote on an iPad using an Apple Pencil. (**b**) Three sample shapes were displayed on the screen: a spiral, a square wave and a triangular wave. The participants were required to trace precisely along the shape guides.

**Figure 3:**
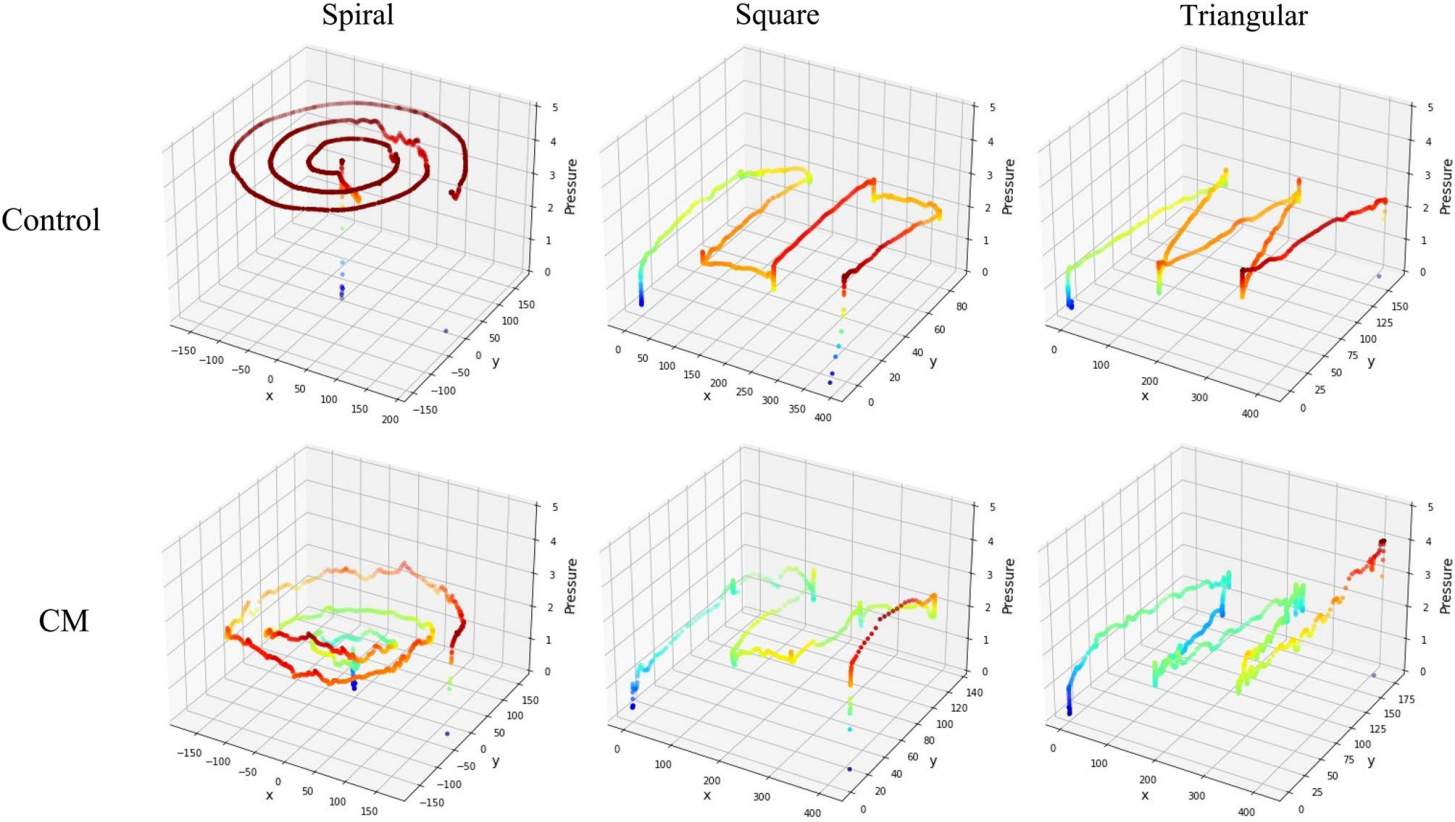
Three-dimensional graphs of pen-tip trajectories drawn by a participant in the non-cervical myelopathy (CM) and the CM group. The x- and y-axes represent coordinates, and the z-axis represents drawing pressure. Note that these graphs only show one of many cases and do not reflect the entire cohort.

We collected data from the participants’ writing motions using a commercially available tablet (iPad Pro 11-inch, Apple, CA, USA) and stylus pen (2nd generation Apple Pencil, Apple, CA, USA), which were widely available at the time (Fig. 2a). Participants were asked to trace three different shapes displayed on the screen with their hands placed on the screen. The shapes included spiral, square, and triangular waves (Fig. 2b). Spiral shapes have been reported to be useful for diagnosing neurological disorders^20–23^, and square and triangular waves are new additions. The participants were instructed to start tracing the spiral from the center and the square and triangular waves from the left side, in one stroke and at their usual speed. While they were drawing, the coordinates, velocity, pressure of the stylus tip, and drawing time were measured (Fig. 3). The tablet screen frame rate was 120 frames per second, and the stylus tip pressure ranged from 0 to 4.166667, which are the default settings in the tablet's operating system. The participants were asked to trace each shape three times; the first two tracings were for practice and the last was used for the data analysis. The tasks were performed in a hospital setting with strictly controlled room temperatures to account for the effect of temperature on hand movements.

### Data analysis

Of the output values that can be measured on the iPad, we focused on the pressure of the stylus tip and drawing time (Table 5). We used average pressure and SPARC, which are features related to writing pressure, to express a quantitative measure of the smoothness of the pressure change^38^. The SPARC indicates the arc length of the Fourier magnitude spectrum of the pressure change, and the larger the SPARC value, the smoother the pressure change. Comparisons between the non-CM and CM groups were conducted for each feature.

**Table 5:** Training features for the support vector machine.

Feature	Feature description
Drawing time (DT)	Time taken to draw each shape
Average pressure (AP)	Average of the writing pressure during the drawing of each shape
SPARC (SP)	Smoothness of the pressure change

SPARC, spectral arc length.

### CM classification using a support vector machine

In this study, we used a two-class classification model with a SVM^39^ to classify non-CM and CM. SVM is a supervised machine learning algorithm for classification and regression analysis, and is commonly used in classification studies because of its high training speed and good accuracy^39^. We adapted the three aforementioned features (drawing time, average pressure, and SPARC) to the SVM to calculate accuracy for each shape. In addition, combinations of features across shapes were also computed. Consequently, seven SVM classification models were created. To generate test data for analysis from the combined variable dataset, we added normalization processing. Leave-one-out cross-validation was used to verify the classification accuracy of the SVM model. Leave-one-out cross-validation, a widely used method to evaluate the performance of classification algorithms, is a cross-validation method in which each dataset is considered the test data and the remaining datasets are training data^40^. To evaluate the accuracy of the classification model, an ROC curve was generated and the AUC was calculated. The hyperparameters for the SVM, such as the regularization parameter, were tuned to maximize the sensitivity and specificity, which meant that the cutoff point would be closest to the upper left of the ROC curve.

### Statistical analysis

We used a two-tailed Student’s t-test to compare age and a chi-square test to compare sex between the non-CM and CM groups. The Mann–Whitney U test was used to compare drawing time, average pressure, and SPARC between the non-CM and CM groups. Statistical significance was set at P < 0.05. All data analyses were performed using Python version 3.9.2 (Python Software Foundation, Delaware, USA).

## Data Availability

The datasets analyzed during the current study are available from the corresponding author on reasonable request.
